# Cardiorenal ketone metabolism: a positron emission tomography study in healthy humans

**DOI:** 10.3389/fphys.2023.1280191

**Published:** 2023-10-06

**Authors:** Bernard Cuenoud, Etienne Croteau, Valérie St-Pierre, Gabriel Richard, Mélanie Fortier, Camille Vandenberghe, André C. Carpentier, Stephen C. Cunnane

**Affiliations:** ^1^ Department of Medicine, Faculty of Medicine and Health Sciences, Université de Sherbrooke, Sherbrooke, Canada; ^2^ Centre D’imagerie Moléculaire de Sherbrooke, Sherbrooke, Canada; ^3^ Centre de Recherche Du CHUS, Sherbrooke, Canada; ^4^ Nestlé Health Science, Lausanne, Switzerland; ^5^ Centre de Recherche sur le Vieillissement, Sherbrooke, Canada

**Keywords:** heart, kidney, ketone, ^11^C-acetoacetate, ^11^C-acetate, cardiorenal, metabolism, beta-hydroxybutyrate

## Abstract

Ketones are alternative energy substrates for the heart and kidney but no studies have investigated their metabolism simultaneously in both organs in humans. The present double tracer positron emission tomography (PET) study evaluated the organ distribution and basal kinetic rates of the radiolabeled ketone, ^11^C-acetoacetate (^11^C-AcAc), in the heart and kidney compared to ^11^C-acetate (^11^C-Ac), which is a well-validated metabolic radiotracer. Both tracers were highly metabolized by the left ventricle and the renal cortex. In the heart, kinetic rates were similar for both tracers. But in the renal cortex, uptake of ^11^C-Ac was higher compared to ^11^C-AcAc, while the reverse was observed for the clearance. Interestingly, infusion of ^11^C-AcAc led to a significantly delayed release of radioactivity in the renal medulla and pelvis, a phenomenon not observed with ^11^C-Ac. This suggests an equilibrium of ^11^C-AcAc with the other ketone, ^11^C-D-beta-hydroxybutyrate, and a different clearance profile. Overall, this suggests that in the kidney, the absorption and metabolism of ^11^C-AcAc is different compared to ^11^C-Ac. This dual tracer PET protocol provides the opportunity to explore the relative importance of ketone metabolism in cardiac and renal diseases, and to improve our mechanistic understanding of new metabolic interventions targeting these two organs.

## 1 Introduction

**FIGURE 1:**
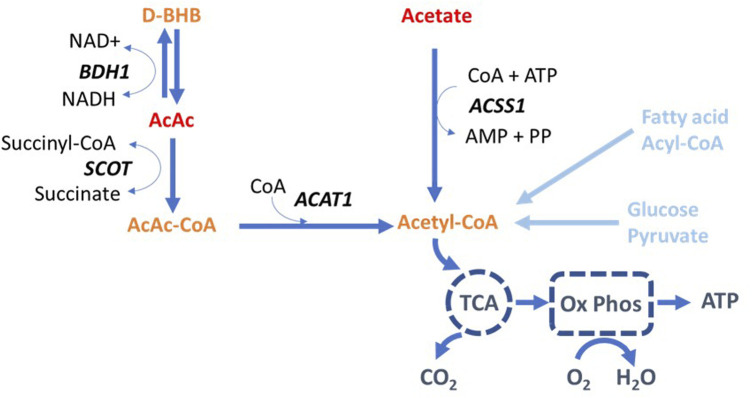
Metabolism of acetoacetate and acetate leading to the production of mitochondrial acetyl-CoA. Fatty acids and glucose are the main precursors of acetyl-CoA via acyl-CoA and pyruvate, respectively. AcAc: acetoacetate; ACAT: acetyl-CoA acetyltransferase; ACSS: acyl-CoA short-chain synthetase; AMP: Adenosine monophosphate; ATP: Adenosine triphosphate; BDH: 3-hydroxybutyrate dehydrogenase; CoA: Coenzyme A; D-BHB: D-beta-hydroxybutyrate; NAD: Nicotinamide adenine dinucleotide; PP: pyrophosphate; Ox Phos: oxidative phosphorylation; SCOT: succinyl-CoA:3-ketoacid CoA transferase; TCA: tricarboxylic acid cycle (or Krebs’s cycle).

Positron emission tomography (PET) is a sensitive technique to study physiology, metabolism, and molecular pathways in living humans using labeled endogenous and natural exogenous compounds (Tersalvi et al., 2023; Toyama et al., 2021; Amoabeng et al., 2022). The ketone radiotracer ^11^C-acetoacetate (^11^C-AcAc) was developed to assess brain ketone metabolism in conditions such as mild cognitive impairment and Alzheimer’s disease (Croteau et al., 2018), which are characterized by regional brain glucose hypometabolism but with preserved brain ketone metabolism (Cunnane et al., 2020). Once injected, AcAc equilibrates rapidly with D-BHB (Figure 1) and is transformed into AcAc-CoA and acetyl-CoA in the mitochondria of key organs such as the brain (Matsuura et al., 2023). Acetyl-CoA enters the Krebs’s (Tricarboxylic Acid [TCA]) cycle as a key substrate leading to the production of energy and ATP via the oxidation-phosphorylation pathway. PET with ^11^C-AcAc also allows increased brain metabolism of ketones from an exogenous ketone supplement to be measured (Fortier et al., 2019). Recently, a pilot PET study with ^11^C-AcAc confirmed that the heart and kidney actively consume exogenous D-beta-hydroxybutyrate (D-BHB), with uptake significantly greater than that of the brain (Cuenoud et al., 2020).

The PET tracer, ^11^C-acetate (^11^C-Ac), has been used extensively to measure oxidative metabolism and substrate perfusion, both in the heart and the kidney (Klein et al., 2001; Normand et al., 2019). In the heart, ^11^C-Ac is rapidly taken up by the left ventricle reflecting the TCA cycle flux well (Figure 1). It provides a good estimate of both myocardial oxygen consumption (MVO_2_) using the tracer clearance rate, and myocardial blood flow (MBF) determined from the tracer uptake rate (Klein et al., 2001). Both values are important to understand various pathophysiological mechanisms and are directly related to cardiac function. In the kidney, rapid uptake of ^11^C-Ac is observed exclusively in the cortex, with minimal medullary uptake or pelvic urinary excretion, and with a clearance rate similar to its myocardial clearance relative to the oxygen consumption index (Juillard et al., 2007; Normand et al., 2019). In the kidney with impaired function, both ^11^C-Ac uptake and clearance are reportedly decreased (Shreve et al., 1995).

In the present study, we performed a head-to-head comparison of ^11^C-AcAc and ^11^C-Ac using dynamic imaging on a high-resolution PET scanner with a large-field of view in which the human heart and kidney could be viewed simultaneously. We determined organ distribution and basal kinetic rates of absorption and metabolism for both tracers.

## 2 Materials and methods

### 2.1 Study approval

The study protocol was approved by the Human Ethics Committee of the CIUSSS-Estrie-CHUS. All participants provided written informed consent in accordance with the Declaration of Helsinki. This study was registered at ClinicalTrials.gov (NCT05238805) under the name *Double* Pet Project: A comparison between ^11^C-acetate and ^11^C-acetoacetate heart and kidney uptake.

### 2.2 Experimental design

#### 2.2.1 Participant preparation and imaging

Ten healthy participants (26.3 ± 3.7 years old, BMI 24.1 ± 4.3 kg/m^2^, see Supplementary material for full characterization) were asked to come for a single session during which the ^11^C-AcAc and ^11^C-Ac PET scans were performed consecutively within 2 h at to ensure that all participants were imaged under similar conditions, they were asked to refrain from taking alcohol or performing intense physical activity 24 h beforehand. To standardize the cohort and evaluate the two radiotracers at baseline without elevated blood ketones, they were asked to be fasted for 4 h and to drink 500 mL of water 1 h before imaging. A blood sample for clinical chemistry was taken immediately before starting the first scan. Each cardiac-gated, list-mode scan started at the time of intravenous injection of the radiotracer (319 ± 53 MBq) and lasted 30 min. There was a 30-min wash-out period between the two tracers, which were administered in random order. Both radiotracers were labelled at carbon position one, with >98% of purity, and specific activity >18.4 GBq/µmol for ^11^C-Ac and >158.4 GBq/µmol for ^11^C-AcAc. Blood pressure and heart rate were monitored at baseline and throughout each scan.

#### 2.2.2 Imaging protocol and image reconstruction

All PET scans were performed on a Biograph Vision 600 (Siemens, Erlangen, Germany) scanner with a 26 cm axial field of view, a matrix of 220 × 220 voxels and a voxel size of 1.65 mm^3^. A 30-min list-mode PET acquisition was performed along with electrocardiogram cardiac gating. Two image reconstructions (dynamic and gated) were performed with an iterative algorithm using point spread function and time of flight modeling. For the dynamic reconstruction, time frames were reconstructed as follows: 12 × 10 s, 6 × 30 s, 6 × 150 s, 2 × 300 s. For the cardiac-gated reconstruction, the sum of the 5–30 min post-injection images was reconstructed into 16 gates.

#### 2.2.3 Image analysis

The pharmacokinetic and left ventricle heart function analyses were performed with PMOD software (PMOD Technologies LLC., Zurich, Switzerland). Right ventricle heart function analyses were performed using Cedars-Sinai Medical Center Cardiac suite software (QPET; Cedars-Sinai, CA, United States). Only the first 15 min of each dynamic scan were used for pharmacokinetic modeling due to the rapid decay of the ^11^C signal. For analysis of heart function with the gated images, the last 25 min of the scan were used to avoid contamination of the images by the tracer in blood.

For cardiac pharmacokinetic analysis, the PMOD cardiac module was used which provides a semi-automatic segmentation of the myocardial regions and ventricles. The same module was used for pharmacokinetic modeling with a heart-specific, one-tissue, two-compartment model with built-in corrections for partial-volume and blood metabolites. The arterial input function was derived from the left ventricle region. For ^11^C-Ac, plasma metabolite correction was performed using values from the literature and built into the PMOD cardiac module (van den Hoff et al., 2001). For ^11^C-AcAc, plasma ^11^C-CO_2_ concentration was measured in a separate cohort of healthy participants and a linear model correction was applied with respect to the timeline acquisition ([^11^C-CO_2_] = 1.323*t) (Supplementary Figure S1). The pharmacokinetic parameters provided *K*_1_ (mL/g/min), representing the radiotracer uptake by the tissue (perfusion), and *k*_2_ (min^-1^) representing tracer rapid clearance or metabolism into ^11^CO_2_. MBF and MVO_2_ were calculated based on the ^11^C-Ac pharmacokinetic parameters (van den Hoff et al., 2001). The relationship between *K*_*1*_ of ^11^C-AcAc and the MBF derived from ^11^C-Ac was investigated using a generalized Renkin-Crone model (van den Hoff et al., 2001). To account for fluctuations of the heart rate and blood pressure between scans, the *K*_1_ was scaled (*K*_*1*_^*s*^) using the rate-pressure product (RPP) with the following formula in which HR is the heart rate, and SBP is the systolic blood pressure:

$$RPP=\left(HR*SBP\right)/1000$$

K1s=K1RPP

Gated cardiac imaging was analyzed from automatic segmentation to obtain the left and right ventricle ejection fraction, end-diastolic volume, and end-systolic volume (Labbé et al., 2012). For the kidney pharmacokinetic analysis, the cortex was segmented manually on a summed image of the radiotracer washout period (2–30 min). Great care was taken to avoid large blood vessels or any contamination of the signal from urine and renal pelvis. We did not attempt to segment the renal medulla because of the high ^11^C signal in the pelvis region following administration of ^11^C-AcAc which was not observed with ^11^C-Ac. A one-tissue, two-compartment model was used for both radiotracers. Additionally, an irreversible two-tissue, three-compartment model was assessed for ^11^C-AcAc. The two-tissue model had a third parameter, *k*_*3*_ representing the local accumulation of ^11^C-AcAc or its metabolites. The one and two tissue models were compared visually and with the Akaike information criterion and were found to be equivalent. The two-tissue model for ^11^C-Ac was not assessed because previous studies by our group showed that the one-tissue model was superior to assess perfusion and oxygen consumption (Bjornstad et al., 2023). The blood input function provided to the model was the same as for the cardiac analysis (left ventricle) with identical correction for the presence of metabolites. Quantification of blood flow or oxygen consumption by the kidney using ^11^C-Ac is not yet fully validated, so only *K*_1_ and *k*_2_ were calculated for both radiotracers (Normand et al., 2019).

#### 2.2.4 Statistical analysis

Statistical analyses were performed using PRISM 9.5.1 (GraphPad Software Inc., CA, United States). All data are expressed as the mean (standard deviation) or median [interquartile range] based on visual inspection of histograms for distribution. Pharmacokinetic data were normally distributed, so statistical differences were investigated using either a paired Student’s t*-*test or a repeated measures one-way ANOVA with Tukey correction for multiple comparisons. Pearson correlation coefficients were also calculated. Heart function parameters following a normal distribution were compared using a paired Student’s *t*-tests, other parameters were compared using a Wilcoxon signed-rank test. A *p*-value ≤0.05 was considered significant.

## 3 Results

**FIGURE 2:**
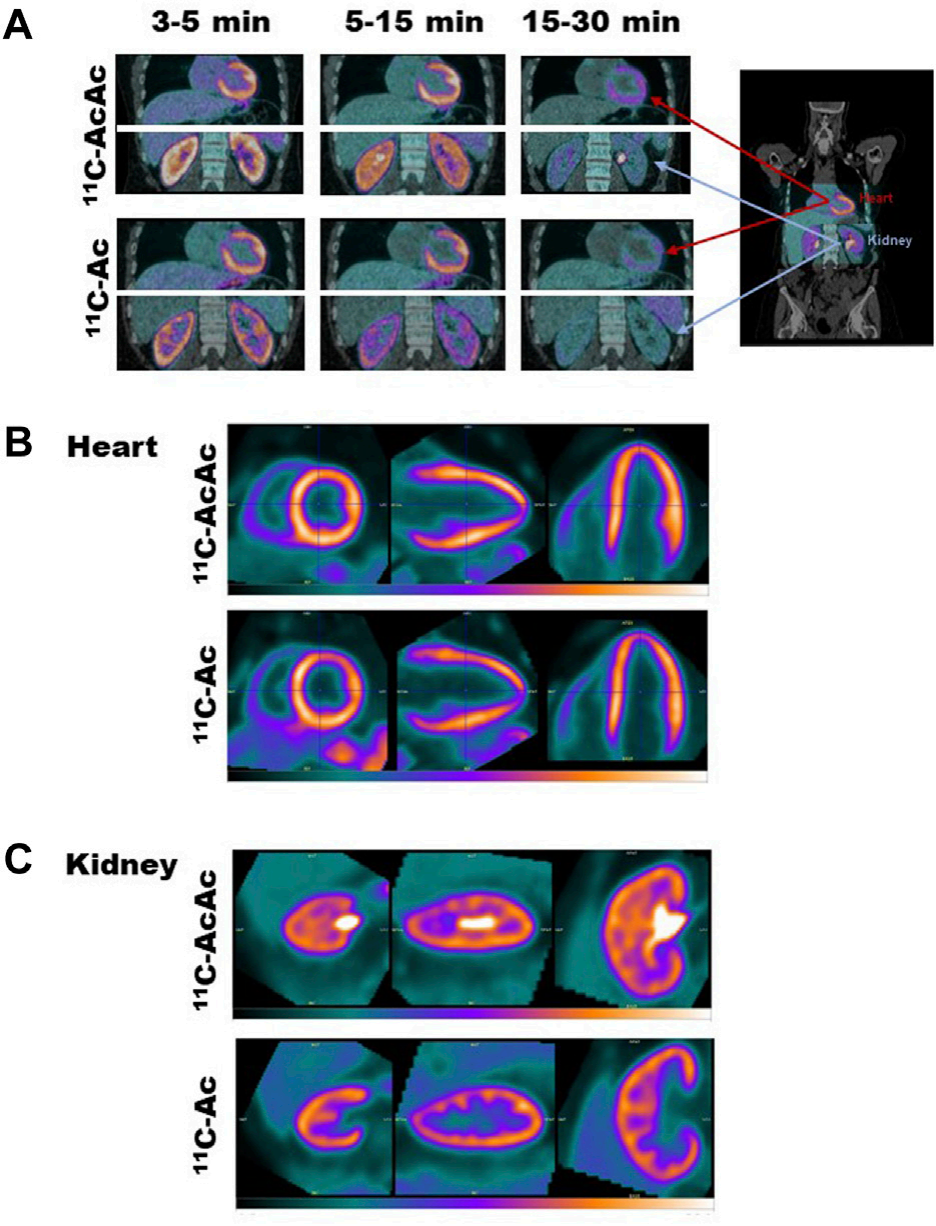
Typical PET images. **(A)** Dynamic time frames after tracer administration for the heart and kidney within the 26-cm axial field of view of the PET scanner superimposed on a low-dose computerized tomography. Summed images for the heart (5–30 min) **(B)** and kidney (5–15 min) **(C)** in the short, vertical, and horizontal long axis for the two radiotracers, ^11^C-AcAc (acetoacetate) and ^11^C-Ac (acetate).

**FIGURE 3:**
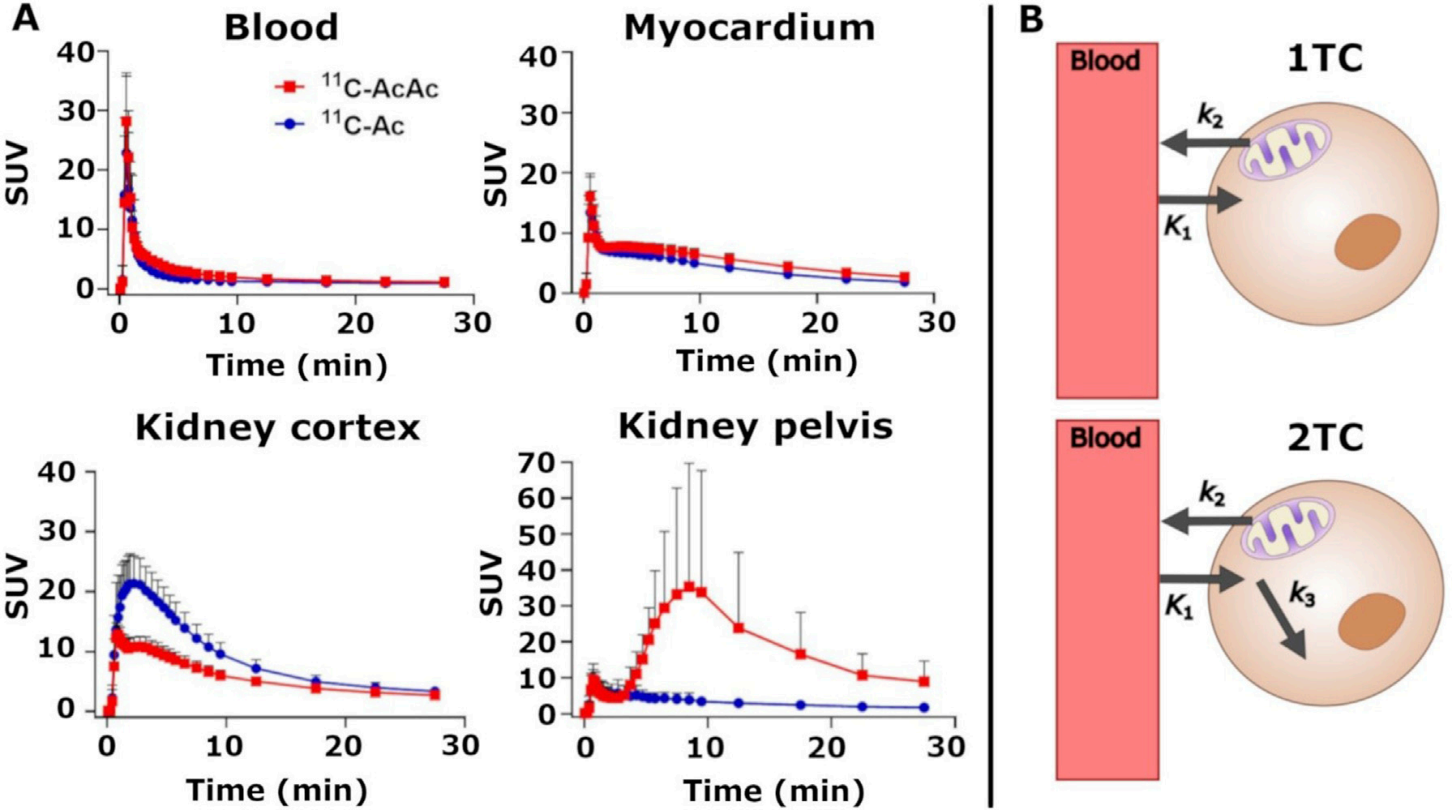
**(A)** ^11^C-AcAc (acetoacetate) and ^11^C-Ac (acetate) time activity curves. Each point is the average ± standard deviation (n = 10). **(B)** Compartmental models were used to derive the pharmacokinetic parameters. 1TC: one-tissue compartment model; 2 TC: two-tissue compartment model; SUV—standardized uptake value.

Figure 2 shows typical images obtained with the two tracers with the heart and kidneys scanned simultaneously. Dynamic time frames after tracer administration superimposed on a low-dose computed tomography (Figure 2A) illustrate the time dependent uptake and clearance of the tracers in the left ventricle and kidney cortex. The heart ^11^C-AcAc and ^11^C-Ac images (Figure 2B) were very similar in appearance with most of the intensity of both tracers located in the left ventricle. The time-activity curves for the two radiotracers were very similar for the left ventricle (Figure 3A), with a very fast uptake, and a similar maximum standardized uptake value (SUV_max_; Table 1). However, for the kidney, the images for the two tracers differed significantly: ^11^C-Ac was distributed mainly in the cortex, while ^11^C-AcAc was also detected in the medulla, renal pelvis and ureter, where it had a hyperintense signal (Figure 2C). In the renal cortex, ^11^C-AcAc was taken up faster than ^11^C-Ac but reached a lower SUV_max_ (Figure 3A). In the renal pelvis, a small initial signal appeared rapidly with both tracers (Figure 3A). A later and larger signal with a T_max_ of 7.1 min appeared only with ^11^C-AcAc (Table 1).

**TABLE 1:** Kinetic analysis of ^11^C-AcAc (acetoacetate) and ^11^C-Ac (acetate) in the heart and kidney. Values are presented as mean (standard deviation). ^11^C-AcAc: ^11^C-acetoacetate; ^11^C-Ac: ^11^C-acetate; SUV_max_: maximum standardized uptake value; T_max_: time to reach SUV_max_; *K*_1_: kinetic rate for tracer transport into the organ; *K*_*1*_^*s*^: kinetic rate for tracer transport into the organ normalized with the rate pressure product; *k*_2_: rate constant for tracer clearance from the organ; MBF: myocardial blood flow; MVO_2_: myocardial oxygen consumption, calculated by the product 1.35 x *k*_2_ -9.6 × 10^−3^; 1 TC: one-tissue compartment model; 2 TC: two-tissue compartment model. *p ≤ 0.05; **p ≤ 0.01; ***p ≤ 0.001 compared to ^11^C-Ac.

		^11^C-AcAc	^11^C-Ac
**Heart Left Ventricle**	SUV_max_	17.7 (1.5)	17.2 (3.0)
	T_max_ (min)	0.58 (0.17)	0.58 (0.21)
	*K*_*1*_ (mL/min/g)—1 TC	0.61 (0.07)**	0.69 (0.10)
	*K*_*1*_^*s*^ (mL/min/g)—1 TC *k*_2_ (min^-1^)—1 TC	0.81 (0.27)*	0.99 (0.22)
		0.063 (0.021)*	0.081 (0.022)
	MBF (mL/min/g)	-	0.80 (0.15)
	MVO_2_ (mL/100 g/min)	-	10.9 (2.9)
**Kidney Cortex**	SUV_max_	14.1 (0.7)***	21.8 (4.5)
	T_max_ (min)	0.75 (0.16)**	2.25 (0.88)
	*K*_*1*_ (mL/min/g) 1 TC	0.83 (0.10)***	1.77 (0.43)
	*K*_*1*_ (mL/min/g) 2 TC	0.87 (0.10)***	-
	*k*_2_ (min^-1^) 1 TC	0.31 (0.04)***	0.21 (0.02)
	*k*_2_ (min^-1^) 2 TC	0.37 (0.06)***	-
	*k*_*3*_ (min^-1^) 2 TC	0.021 (0.015)	-
**Kidney Pelvis**	Sum_ac_	9.94 (3.72)	10.51 (2.74)
	T_max_ (min)	0.75 (0.21)	0.75 (0.75)
	2nd SUV_max_	38.6 (31.5)	-
	2nd T_max_ (min)	6.63 (3.13)	-

### 3.1 Pharmacokinetic analysis of the heart

**FIGURE 4:**
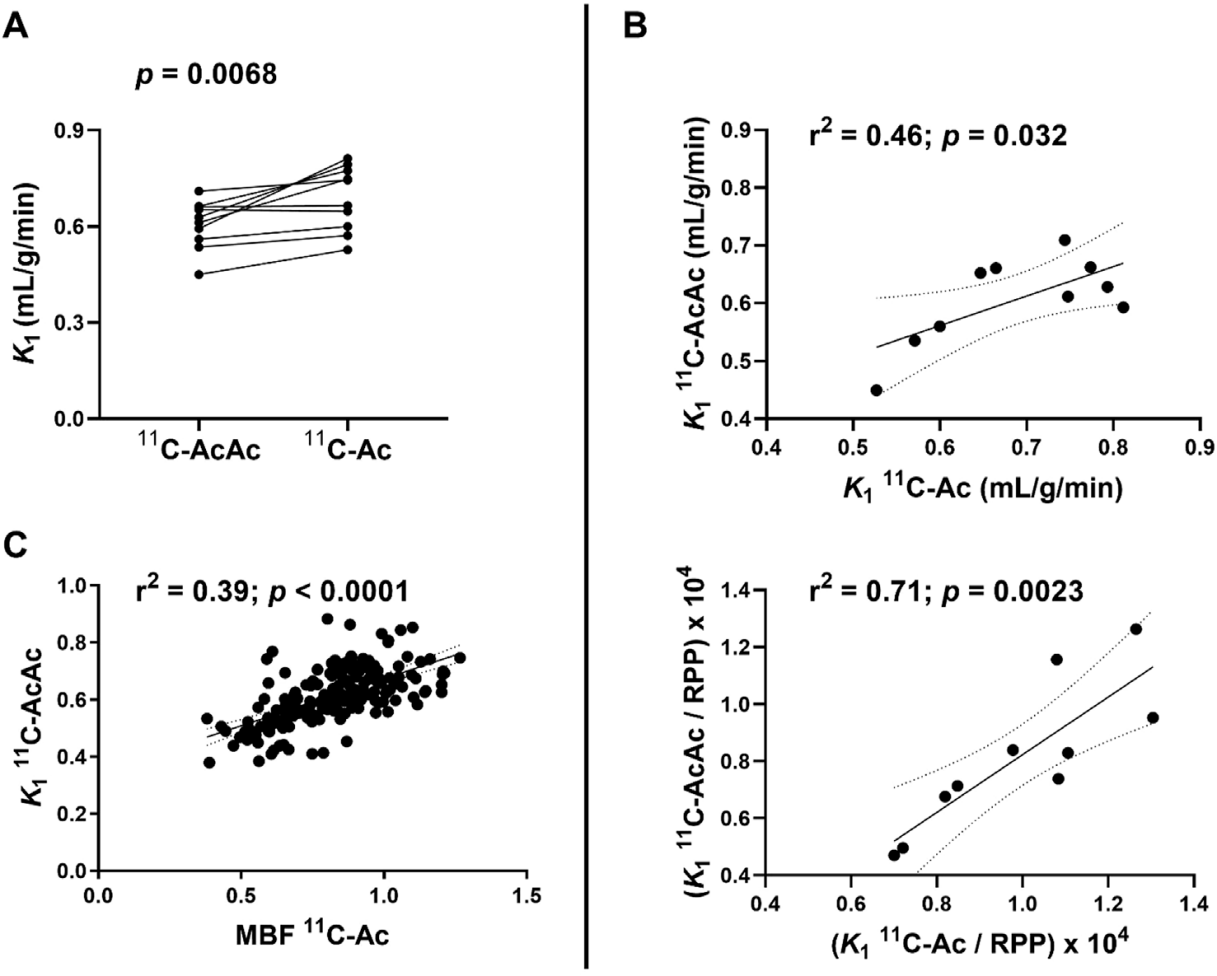
Uptake rate (*K*_1_) of ^11^C-AcAc (acetoacetate) and ^11^C-Ac (acetate) in the myocardium (n = 10). **(A)** Individual *K*_1_ values. Points are paired for both tracers for each participant. **(B)** Correlation of ^11^C-AcAc and ^11^C-Ac *K*_1_ without (upper panel) and with (lower panel) scaling with the individual rate-pressure product (RPP). **(C)** Correlation between ^11^C-Ac derived myocardial blood flow (MBF) and ^11^C-AcAc *K*_1_ for the 17 myocardial segments in all 10 participants. Paired two-tailed Student’s t*-*test for ^11^C-AcAc and ^11^C-Ac. r^2^: coefficient of determination.

**FIGURE 5:**
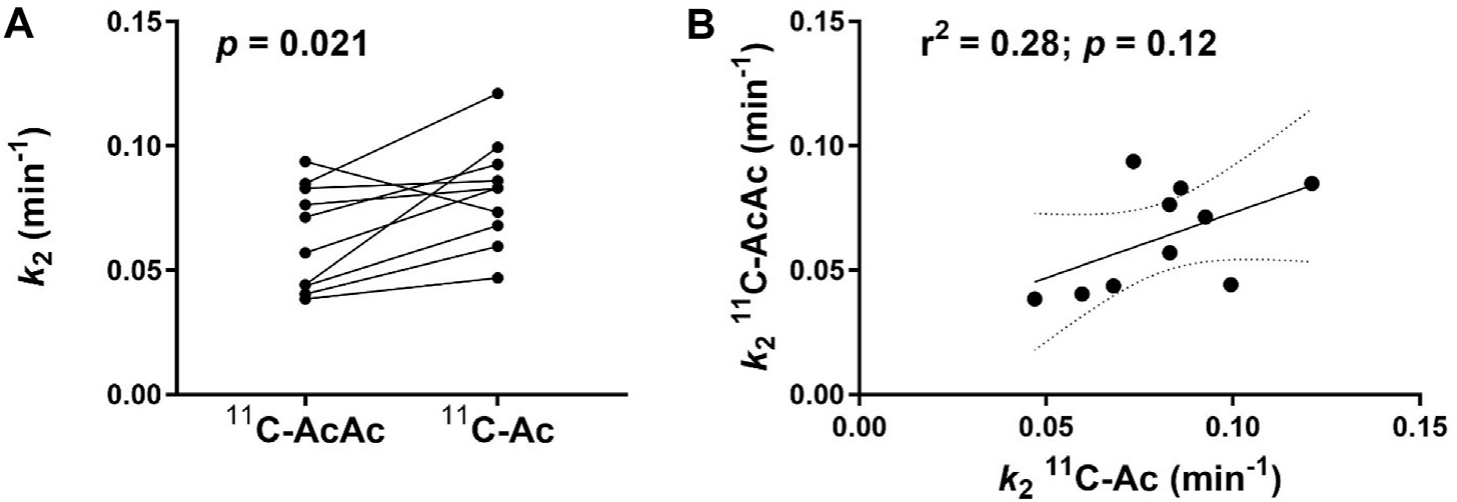
Clearance rate (*k*_2_) of ^11^C-AcAc (acetoacetate) and ^11^C-Ac (acetate) in the myocardium (n = 10). **(A)** Paired data for individual *k*_2_ of ^11^C-AcAc and ^11^C-Ac in each participant. **(B)** Correlation of ^11^C-AcAc and ^11^C-Ac *k*_2_ in each participant. Paired two-tailed Student’s t*-*test for ^11^C-AcAc and ^11^C-Ac. r^2^: coefficient of determination.

Table 1 shows the cardiac pharmacokinetic parameters derived for ^11^C-AcAc and ^11^C-Ac as well as MBF and MVO_2_ for ^11^C-Ac. The values for *K*_1_ and scaled *K*_*1*_ using RPP (*K*_*1*_^*s*^) were similar for both tracers. Figure 4A shows individual *K*_1_ values for the left ventricular myocardium (LV) of each participant. The myocardial *K*_1_ values for the two radiotracers were significantly positively correlated (Figure 4B) prompting us to investigate the possible relationship between MBF derived from ^11^C-Ac and the *K*_1_ of ^11^C-AcAc. Although we found a statistically significant positive correlation between these two parameters (Figure 4C), the narrow range of blood flow values did not allow us to fit a Renkin-Crone model. Scaling the *K*_1_ to the RPP increased the correlation between the two tracers (*r* ^2^ = 0.46 without scaling vs. *r* ^2^ = 0.71 with scaling; Figure 4B), most likely because it accounted for changes in cardiac workload between scans. The cardiac clearance *k*_2_ was higher for ^11^C-Ac compared to ^11^C-AcAc (22%, *p* = 0.021, Figure 5A and Table 1). The *k*_2_s for ^11^C-AcAc and ^11^C-Ac were not significantly correlated (Figure 5B). Heart function parameters and vital signs for both radiotracers are shown in Supplementary Table S2. Vital signs were within normal range and similar during both scans.

### 3.2 Pharmacokinetic analysis of the renal cortex

Table 1 shows the pharmacokinetic parameters obtained for the renal cortex. There was no significant difference between the Akaike information criterion for the one-tissue and irreversible two-tissue model of ^11^C-AcAc. Statistical analyses produced similar results for both models;, accordingly, we present here only results comparing the one-tissue model for ^11^C-AcAc with the one-tissue model for ^11^C-Ac.

**FIGURE 6:**
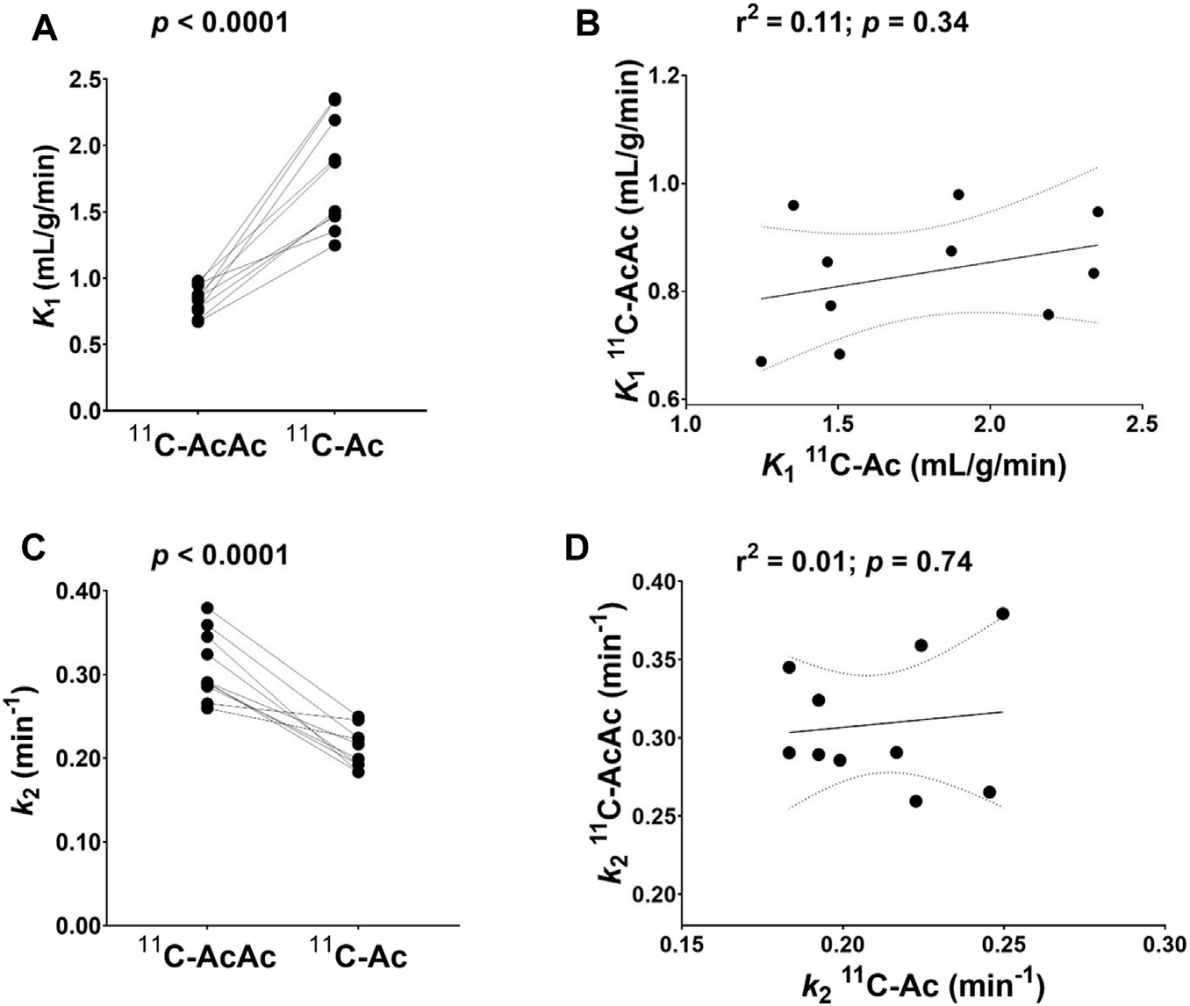
Uptake rate (*K*_1_) and clearance rate (*k*_2_) of ^11^C-AcAc (acetoacetate) and ^11^C-Ac (acetate) in the renal cortex (n = 10) using a one-tissue model. **(A)** Individual *K*_*1*_ values for ^11^C-AcAc and ^11^C-Ac. Points are paired across both tracers for each participant. **(B)** Correlation of ^11^C-AcAc and ^11^C-Ac *K*_*1*_ values with one point for each participant. **(C)** Individual *k*_2_ values for ^11^C-AcAc and ^11^C-Ac. Points are paired across both tracers for each participant. **(D)** Correlation of ^11^C-AcAc and ^11^C-Ac *k*_2_ values with one point for each participant. Paired two-tailed Student’s t*-*test for ^11^C-AcAc and ^11^C-Ac. r^2^: coefficient of determination.

Renal cortical uptake of ^11^C-Ac (*K*_1_) was 53% higher than for ^11^C-AcAc (*p* < 0.0001) (Figure 6A). In contrast to the myocardium, *K*_1_ for ^11^C-AcAc and ^11^C-Ac were not correlated in the renal cortex (Figure 6B). Renal blood flow was not calculated for ^11^C-Ac because it is not yet a fully validated method.

**FIGURE 7:**
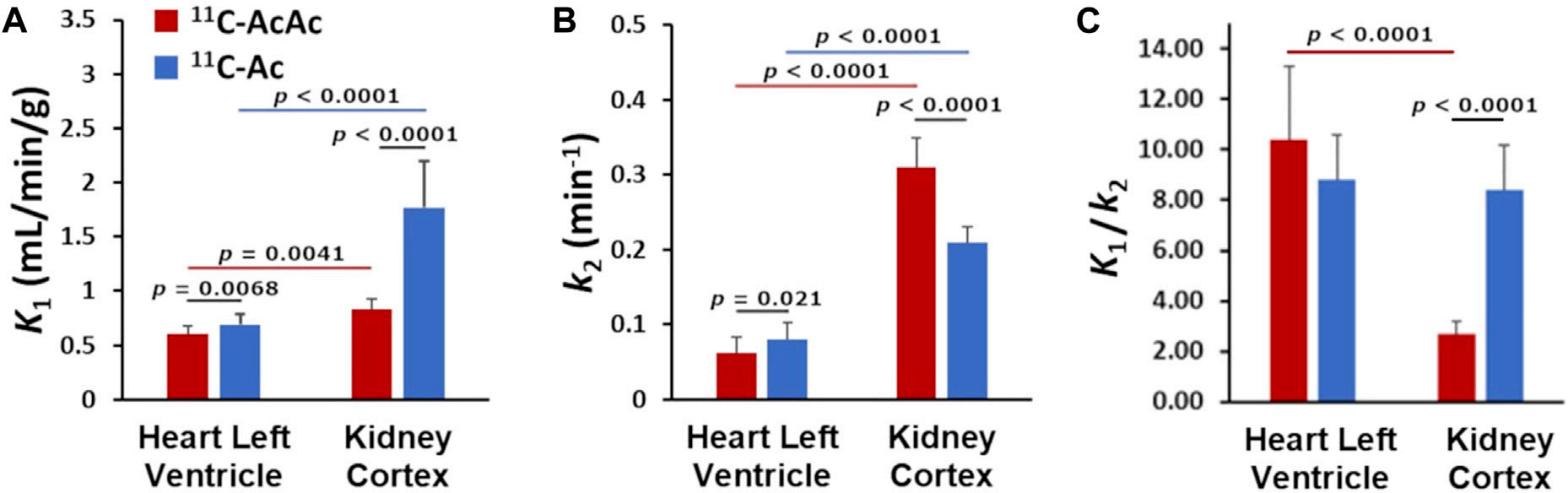
Comparison of ^11^C-AcAc (acetoacetate) and ^11^C-Ac (acetate) kinetic rates in the heart and kidney cortex (n = 10): **(A)** Uptake rate (*K*_*1*_), **(B)** Clearance rate (*k*_*2*_), **(C)** Kinetic ratio *K*_*1*_/*k*_*2.*_

Unlike in the heart, the renal cortex metabolized ^11^C-AcAc at a significantly higher rate than ^11^C-Ac (46%; *p* < 0.0001) (Figure 7C). Similar to *K*_1_, renal *k*_2_ of ^11^C-AcAc and ^11^C-Ac were not significantly correlated (Figure 7D).

### 3.3 Comparison between the kinetic rates of the tracers in the heart versus renal cortex

For both ^11^C-AcAc and ^11^C-Ac, *K*_*1*_ was higher in the renal cortex than in the myocardium (Table 1; Figures 7A, B). Versus the myocardium, *K*_*1*_ for ^11^C-AcAc was 1.4 ± 0.3-fold higher in the renal cortex (*p* = 0.0009) while for ^11^C-Ac, *K*_*1*_ was 2.6 ± 0.8-fold higher in the renal cortex (*p* < 0.0001). Similarly, *k*_*2*_ for ^11^C-AcAc was 5.3 ± 1.6-fold higher (*p* < 0.0001), and for ^11^C-Ac was 2.8 ± 1.0-fold higher (*p* < 0.0001) in renal cortex vs. myocardium.

The *K*_1_/*k*_2_ ratio provides further information about the relationship between tracer uptake and clearance (Figure 7C). This ratio was similar in both organs for ^11^C-Ac, but significantly lower for ^11^C-AcAc in the renal cortex, suggesting the utilization of different kinetic pathways in the kidney.

## 4 Discussion

This study compared the metabolism of the two radiotracers^11^C-AcAc and ^11^C-Ac in the heart and kidney. Similar regional uptake of these tracers was observed with preferential uptake in the left ventricle and in the renal cortex. Overall, the uptake/clearance kinetic ratio (*K*_*1*_/*k*_*2*_) of ^11^C-Ac was similar in the heart and renal cortex, and for ^11^C-AcAc in the heart. However, the kinetic ratio was significantly lower for ^11^C-AcAc the renal cortex (Figure 7C). This suggests that ketones might have similar kinetics as ^11^C-Ac in the heart, but different uptake and clearance in the kidney. Strikingly, ^11^C-AcAc led to a slowly appearing hyperintense signal in the renal pelvis which was not detected with ^11^C-Ac, suggesting additional metabolic pathways possibly involving hepatic ^11^C-AcAc conversion to the other ketone ^11^C-D-BHB. Heart function parameters calculated for each radiotracer provided similar values.

### 4.1 ^11^C-AcAc cardiac PET reveals similar kinetic and functional values compared to ^11^C-Ac

Cardiac PET with ^11^C-Ac is a well-established and validated method to assess MBF and MVO_2_, and our results agree with those already published (Shreve et al., 1995; Normand et al., 2019; Hansen et al., 2022). We compared ^11^C-Ac kinetics to those of ^11^C-AcAc as a first step towards understanding whether the uptake (*K*_*1*_) or clearance (*k*_*2*_) parameters of ^11^C-AcAc were related to blood flow and oxidative metabolism. This information is crucial to correctly interpret dynamic ^11^C-AcAc PET scans assessing cardiac ketone metabolism. Both radiotracers showed relatively high uptake in the myocardium and provide good quality images, with semi-automatic segmentation facilitating the kinetic analysis. Using one compartmental pharmacokinetic modeling, kinetic parameters of ^11^C-AcAc were similar but statistically lower than for ^11^C-Ac. *K*_*1*_swere significantly positively correlated between the two radiotracers; this positive correlation was more evident using a scaled *K*_*1*_ (*K*_*1*_^*s*^; derived via RPP) which took into account the differences in heart rate and blood pressure between participants.

Overall, these results show that, like for ^11^C-Ac, MBF is a determinant of ^11^C-AcAc uptake in the myocardium and that oxidative metabolism is a determinant of its rapid clearance. Comparison between ^11^C-AcAc and ^11^C-Ac in the healthy rat heart also indicates similar kinetics, with those derived from ^11^C-AcAc being 10%–30% lower than with ^11^C-Ac (Croteau et al., 2014), an effect similar what we observed here. The lower myocardial clearance rate of ^11^C-AcAc versus ^11^C-Ac may be attributable to the additional metabolic step required to convert AcAc to acetyl-CoA, and to the equilibrium between AcAc and D-BHB (Figure 1). For heart studies, it is reasonable to assume that the *K*_1_ and *k*_2_ parameters of the one-tissue, two-compartment ^11^C-AcAc model are good indices of tissue perfusion and the rate of ketone metabolism, respectively. However, the exact relationship between *K*_1_ of ^11^C-AcAc and MBF requires further investigation in conditions under which MBF is increased (e.g., stress test) or when the ketone transport rate could be different (e.g., increased ketosis, disease states).

### 4.2 Renal ^11^C-AcAc kinetics are different when compared to ^11^C-Ac

In the kidney, we were able to semi-automatically segment the cortex. Plots of calculated SUV as time activity curves allowed for the kinetic rates of perfusion (*K*_*1*_) and metabolism (*k*_*2*_) of both tracers to be assessed in the renal cortex using a one-tissue two-compartment model and a two-tissue three-compartment model for ^11^C-AcAc. Similar *K*_*1*_ and *k*_*2*_ values were obtained using either one or two-tissue compartment modeling with ^11^C-AcAc. The relatively small *k*_*3*_ value derived from the two-compartment value suggests a low accumulation of ^11^C-AcAc or its metabolite ^11^C-D-BHB in the renal cortex. Therefore, we used the simpler one-tissue model for further comparisons between ^11^C-Ac and ^11^C-AcAc tissue kinetics. However, by neglecting metabolite recirculation and urinary excretion, this model could induce a bias in the calculated ketone consumption rate. Validation of the renal excretion pathway and the metabolic fate of acetoacetate would lead to a more appropriate interpretation of the kinetic analysis results. Moreover, the one-tissue compartment model does not take into account the possibility that circulating ^11^C- CO_2_ could be taken by the tissue. Considering that ^11^C-Ac produces more ^11^C-CO_2_ over the scan period than ^11^C-AcAc, this could explain some of the differences between the two radiotracers. However, most of the uptake and metabolism occur early after radiotracer injection, when blood ^11^C-CO_2_ is low. Also, previous studies (Ng et al., 1994) have described the fate of the ^11^C metabolites in 6 compartments to validate ^11^C-Ac perfusion and oxygen consumption measures.

The use of ^11^C-Ac to assess energy metabolism in the kidney is less well known than for the heart. Direct comparison with ^15^O-H_2_O has shown that uptake of ^11^C-Ac is related to kidney perfusion (Normand et al., 2019). Impaired renal clearance of ^11^C-Ac is a good marker of diabetic nephropathy as well as renal stenosis and is positively correlated to renal oxygen consumption over a wide range of values (Shreve et al., 1995; Juillard et al., 2007). Both uptake and clearance of ^11^C-Ac are higher in the kidney than in the myocardium although the ratio of uptake to clearance (*K*_1_/*k*_2_) remained the same (Figure 7-C).

The kinetics of ^11^C-AcAc in the kidney were quite different to that of ^11^C-Ac. Firstly, ^11^C-AcAc *K*_*1*_ was significantly lower than ^11^C-Ac *K*_*1*_ and only marginally higher than in the myocardium (Table 1; Figures 7–A). This was despite a 3-fold higher blood flow in the renal cortex compared to the myocardium (Normand et al., 2019), which suggests that renal ^11^C-AcAc *K*_*1*_ underestimates renal perfusion to a greater extent than myocardial perfusion. Blood filtrate transit, excretion, and reabsorption in the tubule add a layer of complexity to kidney metabolism that is not present in the myocardium. This could impact measures of renal uptake and metabolism for the two radiotracers and may be sensitive to changes in transport between the red blood cells and plasma, further investigation is needed. Secondly, the clearance *k*_*2*_ of ^11^C-AcAc was significantly higher in the renal cortex compared to the myocardium or to the ^11^C-Ac *k*_*2*_ value. This was striking given the relatively lower ^11^C-AcAc *K*_*1*_ in the renal cortex and suggests longer retention of the ^11^C-Ac in the renal cortex. Overall, these results are consistent with the hypothesis that uptake of ^11^C-AcAc depends more on active transport into kidney cells, with significant irreversible trapping and/or metabolism of ^11^C-AcAc, whereas the kinetic of ^11^C-Ac in the renal cortex, like in the heart, is more dependent on blood flow and better reflects oxygen consumption.

### 4.3 ^11^C-AcAc hyperintensity in the renal pelvis might be derived from ^11^C-D-BHB

A significant part of the signal for ^11^C-AcAc in the kidney was observed in the pelvis and to some degree in the medulla, which was not the case for ^11^C-Ac (Figure 2B). With ^11^C-AcAc, an initial small and rapid increase in the ^11^C-AcAc signal occurred in the renal pelvis was followed by a slower higher intensity signal (Figure 3A). We speculate that this larger second signal was derived from the slower systemic formation and efflux of ^11^C-D-BHB. First, upon intra peritoneal injection of AcAc in mice, a relatively rapid but delayed increase in plasma D-BHB occurs within the first 15–30 min via hepatic BDH-1 metabolism and other BDH-1 expressing tissues (Stagg et al., 2021), suggesting that part of an i.v. dose of ^11^C-AcAc would be rapidly converted to ^11^C-D-BHB. Second, only one rapid 11C-AcAc peak was observed in the renal cortex (Figure 3B). No other peak was detected later, either with ^11^C-AcAc or ^11^C-Ac, and the increase in plasma ^11^C-CO_2_ over time was linear over 30 min (see Supplementary Figure S1). This suggests that the renal pelvic signal could not have originated from a delayed release of ^11^C-AcAc, that it was specific to ^11^C-AcAc and that it was not a downstream metabolite of Ac-CoA. Third, while ketones and Ac are efficiently reabsorbed from the urine by several monocarboxylate transporters localized along the nephron (Becker et al., 2010), subtle differences in transport efficiency between D-BHB and AcAc have been observed and might explain some of the difference in their regional renal reabsorption (Plata et al., 2007). Fourth, in a very recent human PET tracer study (Luong et al., 2023), ^11^C-D-BHB was qualitatively distributed evenly across the entire kidney and was not preferentially retained in the renal cortex, with a rapid constant elimination curve without a second peak. We interpret this to imply that ^11^C-AcAc injected i.v. is probably converted in part to ^11^C-D-BHB and that BHB and AcAc have slightly different regional renal reabsorption and metabolism along the nephron.

### 4.4 Limitations

The current study was performed in healthy participants after a 4-h fast without ketone supplementation, and cardiac PET was performed at rest only. Further studies will be required to evaluate the effect of cardiac stress and circulating energy substrates, especially ketones, on the kinetics of ^11^C-AcAc in diseases such as cardiorenal syndromes and chronic kidney disease. Findings with ^11^C-AcAc in the kidney and the possible role of ^11^C-D-BHB require additional experiments to better understand their regional renal uptake and metabolism. The availability of ^11^C-D-BHB as a new ketone tracer should make these investigations possible.

## 5 Conclusion

This study demonstrates the feasibility of simultaneously measuring heart and kidney ketone metabolism with ^11^C-AcAc in comparison with the metabolic tracer, ^11^C-Ac. Compared to ^11^C-Ac, the most significant difference was detected in the renal cortex, where a slower uptake and faster clearance of ^11^C-AcAc was observed, as well as a slowly appearing hyperintense signal in the renal pelvis. Overall, this suggests that in the kidney, the absorption and metabolism of ^11^C-AcAc is different to that of ^11^C-Ac, in part due to its conversion to ^11^C-D-BHB. Our dual tracer PET protocol provides an opportunity to explore the relative importance of ketone metabolism in cardiac and renal diseases, and to improve our mechanistic understanding of new metabolic interventions.

## Data Availability

The original contributions presented in the study are included in the article/Supplementary Material, further inquiries can be directed to the corresponding author.
