# Supplementary material for: Cardiorenal ketone metabolism: a positron emission tomography study in healthy humans
Source: Front Physiol. 2023 Oct 6;14:1280191. doi: 10.3389/fphys.2023.1280191 (PMC10587428; doi:10.3389/fphys.2023.1280191)
Supplement: Supplementary file 1 [file DataSheet1.pdf]

Supplementary Material

Cardiorenal Ketone Metabolism: a positron emission tomography study in healthy humans

Bernard Cuenoud[1,2,3,4], Etienne Croteau[2,3], Valérie St-Pierre[5], Gabriel Richard[2,3], Mélanie Fortier[5] Camille Vandenberghe[5], André C. Carpentier[1,3], Stephen C. Cunnane[1,5]

[1]Department of Medicine, Faculty of Medicine and Health Sciences, Université de Sherbrooke, Canada

[2]Centre d'imagerie moléculaire de Sherbrooke, Canada

[3]Centre de recherche du CHUS, Canada

[4]Nestlé Health Science, Lausanne, Switzerland

[5]Centre de recherche sur le vieillissement, Sherbrooke, Canada

*** Correspondence:**
Bernard Cuenoud
bernard.cuenoud@nestle.com

1 Participants and clinical chemistry

Ten healthy participants were recruited (Table S1). Inclusion criteria were age (18-55 years old), and BMI (18.5-29 kg/m2). Exclusion criteria were: any medication known to influence energy metabolism, smoking, hypertension, presence of diabetes (fasting glucose >7 mM, or glycosylated hemoglobin >6.5%), intensive physical training or sports program, consuming a ketogenic diet, intermittent fasting, energy restriction or clinically abnormal blood screen.

Blood samples were assayed at the biochemistry core laboratory of the CHUS, except for plasma D-BHB and AcAc collected during the PET scans which were analyzed by automated colorimetric assay on a clinical chemistry analyser (Dimension Xpand Plus; Siemens, Deerfield, IL, USA). Albumin, aspartate aminotransferase, alanine aminotransferase, creatinine, and high- and low-density lipoprotein cholesterol (Roche Diagnostic, Indianapolis, USA) were measured by commercially available kits on an automated analyser (COBAS; Roche Diagnostics, Indianapolis, USA). Glycated hemoglobin was measured by HPLC-723G7, a fully automated high-performance liquid chromatography instrument-reagent system (Tosoh Bioscience, King of Prussia, PA, USA). TSH was measured by sandwich electro-chemiluminescence immunochemistry.

Table S1. Characteristics of the participants. Values are presented as mean ± SD. BMI; Body mass index, GFR; Glomerular filtration rate, BP; Blood Pressure

Male/female (n=10)	3/7		
Age (y)	26.3	±	3.7
BMI (kg/m^2)	24.1	±	4.3
Glucose (mM)	4.8	±	0.5
Acetoacetate (µM)	25.9	±	17.2
B-Hydroxybutyrate (µM)	57.8	±	42.7
GFR (mL/min)	105.4	±	10.2
Vital Signs (baseline)			
Heart Rate (bpm)	70.1	±	13.6
Diastolic BP (mm Hg)	66.8	±	8.4
Systolic BP (mm Hg)	110.0	±	12.7

2 Plasma ^{11}C-CO_2 concentration after ^{11}C-AcAc injection

Plasma ^{11}C-CO_2 concentration was measured in a separate healthy cohort (n=7, mean: 57 year old, 3F/4H, BMI 25) and a linear model correction was derived in regard to the timeline acquisition ([^{11}C-CO_2] = 1.323*t) (Figure S1; (1)).

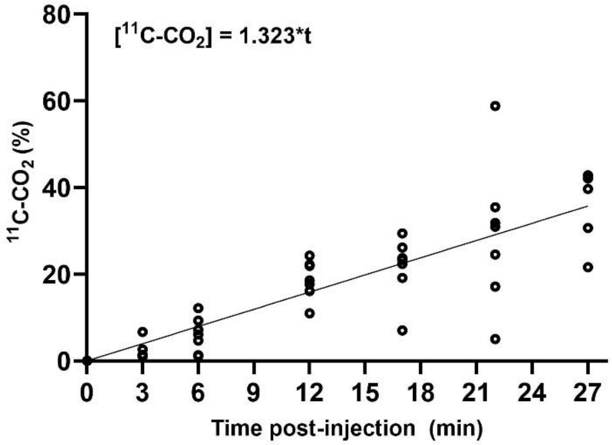

Figure S1. Blood ^{11}C-CO_2 after injection of ^{11}C-AcAc (acetoacetate) in fasting individuals (n = 7).

3 Heart function parameters determined using f^{11}C-AcAc and ^{11}C-Ac

Left ventricle end-diastolic volume was 7% lower for ^{11}C-AcAc compared to ^{11}C-Ac (p = 0.039). End-systolic volumes were not significantly different when measured using each of the two tracers, but a lower end-diastolic volume was observed for ^{11}C-AcAc (p = 0.038).

Table S2. Parameters of heart function. Values are presented as mean (standard deviation) for normally distributed data, and median [interquartile range] otherwise. BP: Blood Pressure. *$p \leq 0.05$ compared to ^{11}C-acetate.

	^{11}C-Acetoacetate	^{11}C-Acetate
Left ventricle		
Ejection fraction (%)	67.2 (9.0)	67.2 (7.6)
End-diastolic volume (mL)	89.0 [56.5]*	102.5 [56.3]
End-systolic volume (mL)	35.3 (19.8)	38.5 (16.3)
Myocardial mass (g)	141.8 (22.2)	143.1 (20.3)
Right ventricle		
Ejection fraction (%)	67.8 (6.8)	67.5 (11.0)
End-diastolic volume (mL)	88.0 [52.5]*	108.0 [52.3]
End-systolic volume (mL)	26.0 [33.3]	33.5 [42.3]
Vital Signs		
Heart rate (bpm)	66.8 (11.3)	69.4 (12.9)
Diastolic BP (mm Hg)	65.2 (7.9)	68.2 (8.5)
Systolic BP (mm Hg)	108.9 (10.0)	111.4 (11.8)
Rate pressure product (heart rate x systolic BP)	7.3 (1.9)	7.8 (2.2)

4 References:

1. Ng Y, Moberly SP, Mather KJ, Brown-Proctor C, Hutchins GD, Green MA. Equivalence of arterial and venous blood for [11C]CO2-metabolite analysis following intravenous administration of 1-[11C]acetate and 1-[11C]palmitate. Nucl Med Biol. 2013;40(3):361-365.

2. Buck A, Wolpers HG, Hutchins GD, et al. Effect of carbon-11-acetate recirculation on estimates of myocardial oxygen consumption by PET. J Nucl Med. 1991;32(10):1950-1957.
